# Early detection of respiratory disease outbreaks through primary healthcare data

**DOI:** 10.7189/jogh.13.04124

**Published:** 2023-11-03

**Authors:** Thiago Cerqueira-Silva, Izabel Marcilio, Vinicius de Araújo Oliveira, Pilar Tavares Veras Florentino, Gerson O Penna, Pablo I Pereira Ramos, Viviane S Boaventura, Manoel Barral-Netto

**Affiliations:** 1 Laboratório de Medicina e Saúde Pública de Precisão – Instituto Gonçalo Moniz, Salvador, Bahia, Brazil; 2 Centro de Integração de Dados e Conhecimentos para Saúde – Instituto Gonçalo Moniz, Salvador, Bahia, Brazil; 3 Centro de Medicina Tropical – Universidade de Brasília, Escola Fiocruz de Governo, Brasília, Brazil; 4 Faculdade de Medicina da Bahia, Universidade Federal da Bahia, Salvador, Brazil

## Abstract

**Background:**

The emergence of coronavirus disease 2019 (COVID-19) in 2020 highlighted the relevance of surveillance systems in detecting early signs of potential outbreaks, thus enabling public health authorities to act before the pathogen becomes widespread. Syndromic digital surveillance through web applications has played a crucial role in monitoring the spread of the severe acute respiratory syndrome coronavirus 2 (SARS-CoV-2) virus. However, this approach requires expensive infrastructure, which is not available in developing countries. Pre-existing sources of information, such as encounters in primary health care (PHC), can provide valuable data for a syndromic surveillance system. Here we evaluated the utility of PHC data to identify early warning signals of the first COVID-19 outbreak in Bahia-Brazil in 2020.

**Methods:**

We compared the weekly counts of PHC encounters due to respiratory complaints and the number of COVID-19 cases in 2020 in Bahia State – Brazil. We used the data from December 2016 to December 2019 to predict the expected number of encounters in 2020. We analysed data aggregated by geographic regions (n = 34) and included those where historical PHC data was available for at least 70% of the population.

**Results:**

Twenty-one out of 34 regions met the inclusion criteria. We observed that notification of COVID-19 cases was preceded by at least two weeks with an excess of encounters of respiratory complaints in 18/21 (86%) of the regions analysed and four weeks or more in 10/21 (48%) regions.

**Conclusions:**

Digital syndromic surveillance systems based on already established PHC databases may add time to preparedness and response to emerging epidemics.

The coronavirus disease 2019 (COVID-19) pandemic highlighted the importance of surveillance systems capable of detecting respiratory outbreaks at early stages, even before pathogen identification. The recent re-emergence of avian influenza A (H5N1), a potential pandemic virus, reinforces the urgency of implementing syndromic tools for the early detection of respiratory diseases. Digital surveillance systems using syndromic data have played a crucial role in monitoring the spread of the severe acute respiratory syndrome coronavirus 2 virus (SARS-CoV-2). Initiatives such as those proposed by the Zoe COVID-19 symptom group and COvid-19: Operation for Personalized Empowerment to Render smart prevention And care seeking (COOPERA) exemplify the high utility of early warning systems [1,2]. However, they require the population’s engagement by accessing the web application and self-reporting information, as well as widespread access to the internet and expensive infrastructures, which limits their application in developing countries.

Here, we present a case study of a syndromic surveillance tool in Brazil that uses digital information routinely collected by the primary healthcare (PHC) system to feed an early warning system.

## METHODS

We used data from the PHC system described in another study [3]. We compared the weekly counts of PHC encounters due to respiratory complaints and the number of COVID-19 cases in 2020 in the state of Bahia, Brazil’s fourth most populous state with 14.9 million inhabitants in 417 municipalities and a territorial extension comparable to France (Figure S1 in the **Online Supplementary Document**). We used the data from December 2016 to December 2019 to predict the number of encounters expected for 2020. We classified encounters coded with any International Classification of Diseases, 10^th^ Revision (ICD-10) or International Classification of Primary Care, 2^nd^ edition (ICPC-2) related to acute respiratory infection (Table S1 in the **Online Supplementary Document**) as encounters due to respiratory complaints. We used the total number of encounters per week as the population offset in the model.

We assessed the completeness of historical data by the number of weeks with a record of any encounters in previous years. For example, a city was considered to have complete historical data if it had at least 155 weeks with PHC records from 31 October 2016, to 29 December 2019 (total of 165 weeks). The cities were aggregated by immediate geographic region (n = 34), as per the Brazilian Institute of Geography and Statistics [4]. We included the regions with more than 70% of the population with complete historical data. We also conducted a similar analysis using the three largest cities in Bahia: Salvador (population = 2 953 986), Feira de Santana (population = 627 477), and Vitória da Conquista (population = 348 718).

### Statistical analysis

We employed the algorithm proposed by Farrington and modified by Noufaliy to estimate the expected number of encounters due to respiratory complaints each week and their respective 95% confidence interval [5]. The upper limit of the 95% confidence interval was defined as the threshold for considering excess cases. The parameters used in the algorithm were three years of historical data using a window half size of three weeks.

We extracted the number of confirmed COVID-19 cases from Bahia’s Secretary of Health public dashboard [6] and employed a piecewise linear regression in the weekly COVID-19 cases in each region to define the onset of the rapid SARS-CoV-2 transmission per region (Figure S5 in the **Online Supplementary Document**).

We extracted the number of hospitalisations due to severe acute respiratory syndrome (SARS) from the *Sistema de Informação de Vigilância Epidemiológica da Gripe* (*SIVER*-*Gripe*) nation-wide surveillance database [7].

We conducted all statistical analyses in R, version 3.6.1 (R Core Team, Vienna, Austria) and its “surveillance”, version 1.20.3 [8] and “segmented” version 1.6.2 [9] packages.

### Ethics

The Bahia State Health Secretary for research purposes provided weekly aggregated data, as authorised in the Brazilian General Protection law. We did not seek ethical approval, as the data could not be re-identified in any way.

## RESULTS

**Figure 1:**
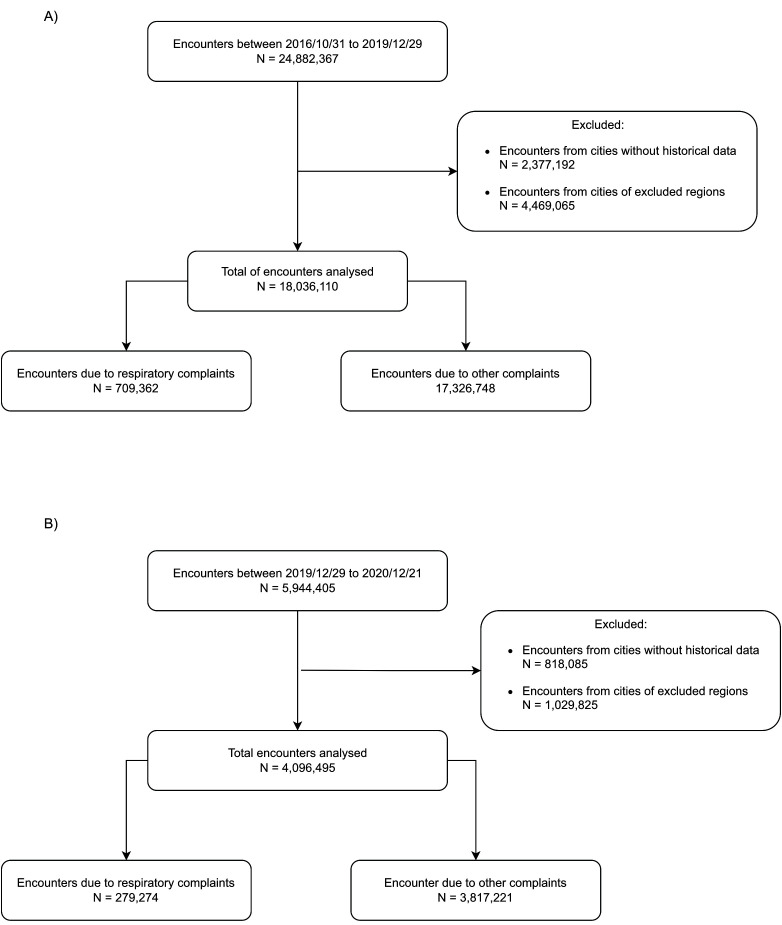
Flowchart of the PHC data analysed. **Panel A.** Baseline. **Panel B.** Test period.

The first COVID-19 case was detected on 25 February 2020 in Brazil and on 6 March 2020 in Bahia. We evaluated a total of 24 882 367 encounters from 2016 to 2019 in 34 immediate geographic regions, 21 (62%) of which met the inclusion criteria (Table S2 in the **Online Supplementary Document**), resulting in 18 036 110 encounters used as baseline data. The validation data were comprised of 4 096 495 encounters that occurred in 2020 (**Figure 1****,** Panels A and B)

**Figure 2:**
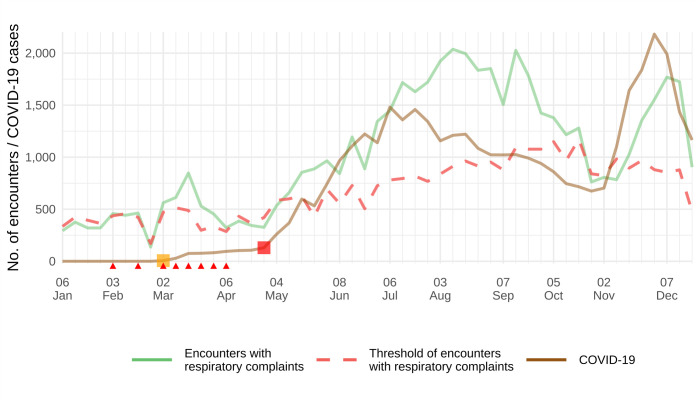
Weekly syndromic surveillance signals from primary health care and weekly COVID-19 cases in the region of Vitoria da Conquista in 2020. The yellow square indicates the week of the first COVID-19 case confirmed in Bahia, the red square indicates the first week of the rapid growth of COVID-19 cases based on piecewise linear regression, and red triangles denote weeks with excess encounters due to respiratory complaints prior to the rapid growth of COVID-19 cases.

We observed an excess of encounters due to respiratory complaints in 18 (86%) regions at least two weeks before the increase in COVID-19 cases, with a median of four weeks (interquartile range (IQR) = 3-6) (Figure S2 in the **Online Supplementary Document**). For example, the region of Vitória da Conquista presented six weeks with an excess of encounters in the nine weeks between the first confirmed COVID-19 case in Bahia and the week of rise in COVID-19 cases in this region (**Figure 2**). Regarding SARS-related hospitalisations, all 21 regions presented COVID-19 hospitalisations after April 2020, coinciding with the pattern of total COVID-19 cases (Figure S4 in the **Online Supplementary Document**).

In the remaining three regions, two presented only one week with excess encounters before the increase in COVID-19 cases, and one did not exhibit any excess encounters due to respiratory complaints before the rise in COVID-19 cases. Analysis of the three largest cities in Bahia showed a minimum of three weeks with an excess of encounters before the rise of COVID-19 (Feira de Santana) and up to seven weeks with an excess of encounters (Vitória da Conquista) (Figure S3 in the **Online Supplementary Document**).

## DISCUSSION

We demonstrate that it is possible to anticipate the rise in respiratory infection cases by analysing routinely collected PHC data in most Bahia State regions. The lack of detection seemed to relate to areas with sparse populations or those underreporting PHC data. This suggests a potential utility of digital health data in early-warning systems for surveillance purposes.

Our findings regarding regions with an excess of PHC encounters due to respiratory complaints prior to the first reported COVID-19 case in Bahia-Brazil are consistent with studies that found evidence of the circulation of SARS-CoV-2 virus two to four weeks in three countries before the first confirmed case by local public health agencies, either using serological surveillance [10,11] or genomic characterisation [12,13]. Only hospitalised patients were reported at the beginning of the COVID-19 pandemic. However, most COVID-19 cases do not require hospitalisation. Since there is a lag of up to two weeks between SARS-CoV-2 infection and progress to severe disease, this situation may also have contributed to the observed excess PHC respiratory-related encounters before the confirmation of the first COVID-19 case. These factors combined hindered the detection of COVID-19 cases in the early period of the pandemic.

In our analysis, the number of encounters in the PHC after the peak of COVID-19 differed greatly by immediate region. One possible reason for this phenomenon is the lack of standardisation in public health recommendations in Brazil during COVID-19 in 2020. The Brazilian Ministry of Health did not develop a national plan in response to the pandemic and did not implement non-pharmacological interventions [14]. Consequently, Brazilian municipalities had to develop individual plans to mitigate the burden of COVID-19, which resulted in heterogeneous and uncoordinated actions [15].

PHC is an integral part of the Brazilian Unified Health System (SUS), which serves as the entrance to healthcare services for all levels of complexity. It is based on family health teams comprising medical doctors, nurse practitioners, nursing assistants, and community healthcare agents. PHC covers at least 74% of the Brazilian population, including the marginalised and those living in remote areas [16]. Despite these characteristics, some factors can interfere with PHC's potential for early detection of future epidemics. One such challenge arises from the lack of data derived from the private healthcare system, which becomes particularly problematic when addressing diseases initially imported from other countries, affecting primarily the more affluent populations who do not use SUS [17] Additionally, concerns persist regarding the potential delays in reporting PHC data, although these delays tend to be shorter than traditional systems relying on laboratory testing [18].

Our study is subject to several limitations. First, we could not link individual PHC encounters and hospitalisation data. This would have allowed us to gain further insights into the number of individuals who progressed to severe cases and their trajectory within the health system. Additionally, our analysis was limited to a single State in Brazil. However, we should note that the State of Bahia is geographically vast and has a highly diverse range of municipalities, with Human Development Index values ranging from 0.5 to 0.75. The early detection in multiple regions of the state provides evidence of the broad applicability and utility of PHC data. This suggests that our approach could be effective across a wide range of situations, further emphasising the potential of PHC data in epidemiological surveillance for early detection of outbreaks.

Digital syndromic surveillance systems based on already established databases, such as the PHC database, may add timeliness to preparedness and response to emerging epidemics. Additionally, implementing such a system could improve the number and quality of PHC data registration, contributing to a structural improvement of the local health system. This strategy is especially advantageous for developing countries that chronically face constrained health resources.

## Additional material


Online Supplementary Document
