# Supplementary material for: Early detection of respiratory disease outbreaks through primary healthcare data
Source: J Glob Health. 2023 Nov 3;13:04124. doi: 10.7189/jogh.13.04124 (PMC10623377; doi:10.7189/jogh.13.04124)

Contents

Table S1: ICD-10 and ICPC-2 codes used to define a respiratory complaint 2

Table S2: Description of Bahia state regions: number of cities, population and Availability of Primary Health Care historical data. 3

Figure S1: Human development index (HDI) and population size from the 417 Bahia's cities. 5

Figure S2: Weekly syndromic surveillance signals from PHC and weekly COVID-19 cases from the selected regions in 2020. The yellow square indicates the week of the first COVID-19 case confirmed in Bahia. The red square indicates the first week of the rapid growth of COVID-19 cases based on piecewise linear regression. The red triangles denote weeks with excess encounters due to respiratory complaints prior to the rapid growth of COVID-19 cases. 6

Figure S3: Weekly syndromic surveillance signals from PHC and weekly COVID-19 cases from the three largest cities in Bahia 2020. The yellow square indicates the week of the first COVID-19 case confirmed in Bahia. The red square indicates the first week of the rapid growth of COVID-19 cases based on piecewise linear regression. The red triangles denote weeks with excess encounters due to respiratory complaints prior to the rapid growth of COVID-19 cases. 9

Figure S4: Fortnight number of hospitalisations from Severe Acute Respiratory Syndrome from the selected regions in 2020 stratified by diagnosis. 10

Figure S5: Example of piecewise regression and COVID-19 cases to define the breakpoints. Red line denotes COVID-19 cases, and the black line is the piecewise linear regression. 12

Table S1: ICD-10 and ICPC-2 codes used to define a respiratory complaint

Type	Code	Description
ICPC - 2	A03	Fever
ICPC - 2	R02	Shortness of breath/dyspnoea
ICPC - 2	R04	Breathing problem, other
ICPC - 2	R05	Cough
ICPC - 2	R07	Sneezing/nasal congestion
ICPC - 2	R08	Nose symptom/complaint other
ICPC - 2	R21	Throat symptom/complaint
ICPC - 2	R23	Voice symptom/complaint
ICPC - 2	R25	Sputum/phlegm abnormal
ICPC - 2	R29	Respiratory symptom/complaint oth.
ICPC - 2	R71	Whooping cough
ICPC - 2	R74	Upper respiratory infection acute
ICPC - 2	R75	Sinusitis acute/chronic
ICPC - 2	R76	Tonsillitis acute
ICPC - 2	R77	Laryngitis/tracheitis acute
ICPC - 2	R80	Influenza
ICPC - 2	R81	Pneumonia
ICPC - 2	R83	Respiratory infection other
ICPC - 2	R99	Respiratory disease other
ICD – 10	J00 - J06	Acute upper respiratory infections
ICD – 10	J09 - J18	Influenza and pneumonia
ICD – 10	J20 - J22	Other acute lower respiratory infections
ICD – 10	R05	Cough
ICD – 10	R06	Abnormalities of breathing
ICD – 10	R07	Pain in throat and chest
ICD – 10	R43	Disturbances of smell and taste
ICD – 10	R50	Fever of other and unknown origin
ICD – 10	U07	Emergency use of U07
ICD – 10	B34	Viral infection of unspecified site
ICD – 10	B97	Viral agents as the cause of diseases classified to other chapters

ICD-10 = International Classification of Diseases 10th Revision

ICPC-2 = International Classification of Primary Care 2nd edition

Table S2: Description of Bahia state regions: number of cities, population and Availability of Primary Health Care historical data.

Bahia Geographic Region	Historical data avalaible	Number of cities	Total of population by group	Population (%)
**Alagoinhas**	No	13	362,336	69.9
	Yes	4	156,276	30.1
**Barreiras**	No	10	280,177	54.9
	Yes	7	230,170	45.1
**Bom Jesus da Lapa**	No	4	187,563	78.5
	Yes	3	51,314	21.5
**Brumado**	No	3	43,314	18.3
	Yes	9	193,893	81.7
**Camacan**	No	2	17,936	12.4
	Yes	6	126,442	87.6
**Conceição do Coité**	No	1	14,730	7.4
	Yes	6	185,021	92.6
**Cruz das Almas**	No	4	87,217	29.4
	Yes	8	209,757	70.6
**Cícero Dantas**	No	1	17,349	13.0
	Yes	5	116,481	87.0
**Euclides da Cunha**	No	1	17,316	8.8
	Yes	4	178,700	91.2
**Eunápolis - Porto Seguro**	No	4	88,419	22.7
	Yes	4	300,457	77.3
**Feira de Santana**	No	14	279,928	22.1
	Yes	19	987,649	77.9
**Guanambi**	No	7	92,327	18.6
	Yes	17	404,448	81.4
**Ilhéus – Itabuna**	No	10	152,572	21.9
	Yes	12	543,398	78.1
**Ipiaú**	No	2	62,416	27.2
	Yes	11	167,399	72.8
**Irecê**	No	10	250,051	59.3
	Yes	9	171,880	40.7
**Itaberaba**	No	3	39,933	17.2
	Yes	9	192,527	82.8
**Itapetinga**	No	3	39,026	24.9
	Yes	3	117,884	75.1
**Jacobina**	No	3	52,505	15.6
	Yes	13	283,460	84.4
**Jequié**	No	5	50,319	13.9
	Yes	11	312,085	86.1
**Jeremoabo**	No	3	69,702	67.1
	Yes	2	34,246	33.0
**Juazeiro**	No	6	441,015	83.1
	Yes	3	89,501	16.9

**Nazaré – Maragogipe**	No	3	47,809	25.7
	Yes	4	138,482	74.3
**Paulo Afonso**	No	2	27,431	13.7
	Yes	5	173,542	86.4
**Ribeira do Pombal**	No	2	78,000	37.3
	Yes	5	131,139	62.7
**Salvador**	No	5	149,061	3.6
	Yes	11	3,950,302	96.4
**Santa Maria da Vitoria**	No	2	52,859	36.6
	Yes	5	91,734	63.4
**Santo Antônio de Jesus**	No	5	59,763	19.8
	Yes	9	242,001	80.2
**Seabra**	No	4	79,675	43.8
	Yes	6	102,171	56.2
**Senhor do Bonfim**	No	3	44,738	14.5
	Yes	6	263,733	85.5
**Serrinha**	No	2	72,588	37.2
	Yes	3	122,762	62.8
**Teixeira de Freitas**	No	6	249,982	53.9
	Yes	7	214,181	46.1
**Valença**	No	5	120,126	46.0
	Yes	3	141,018	54.0
**Vitória da Conquista**	No	11	163,898	19.1
	Yes	19	695,238	80.9
**Xique-Xique – Barra**	No	4	103,366	45.1
	Yes	6	125,731	54.9

Figure S1: Human development index (HDI) and population size from the 417 Bahia's cities.

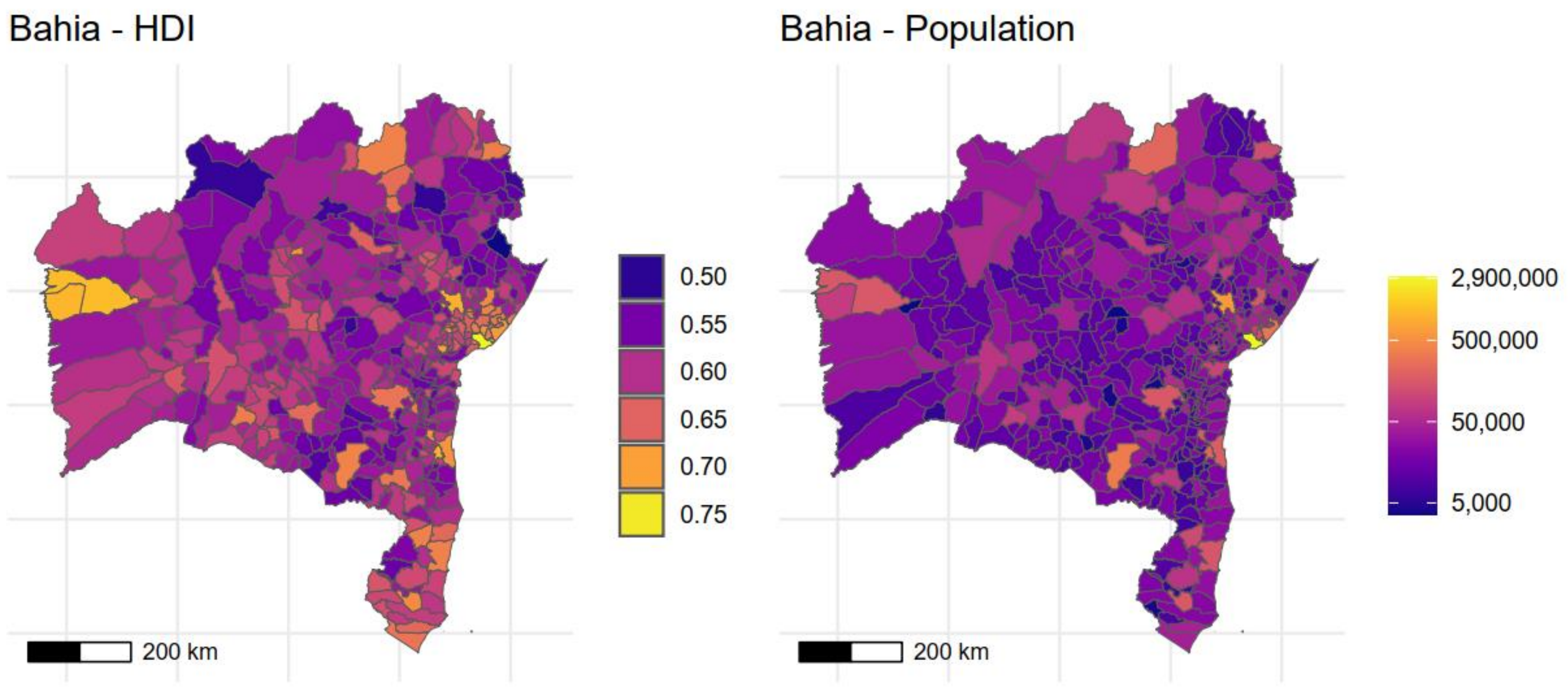

Figure S2: Weekly syndromic surveillance signals from PHC and weekly COVID-19 cases from the selected regions in 2020. The yellow square indicates the week of the first COVID-19 case confirmed in Bahia. The red square indicates the first week of the rapid growth of COVID-19 cases based on piecewise linear regression. The red triangles denote weeks with excess encounters due to respiratory complaints prior to the rapid growth of COVID-19 cases.

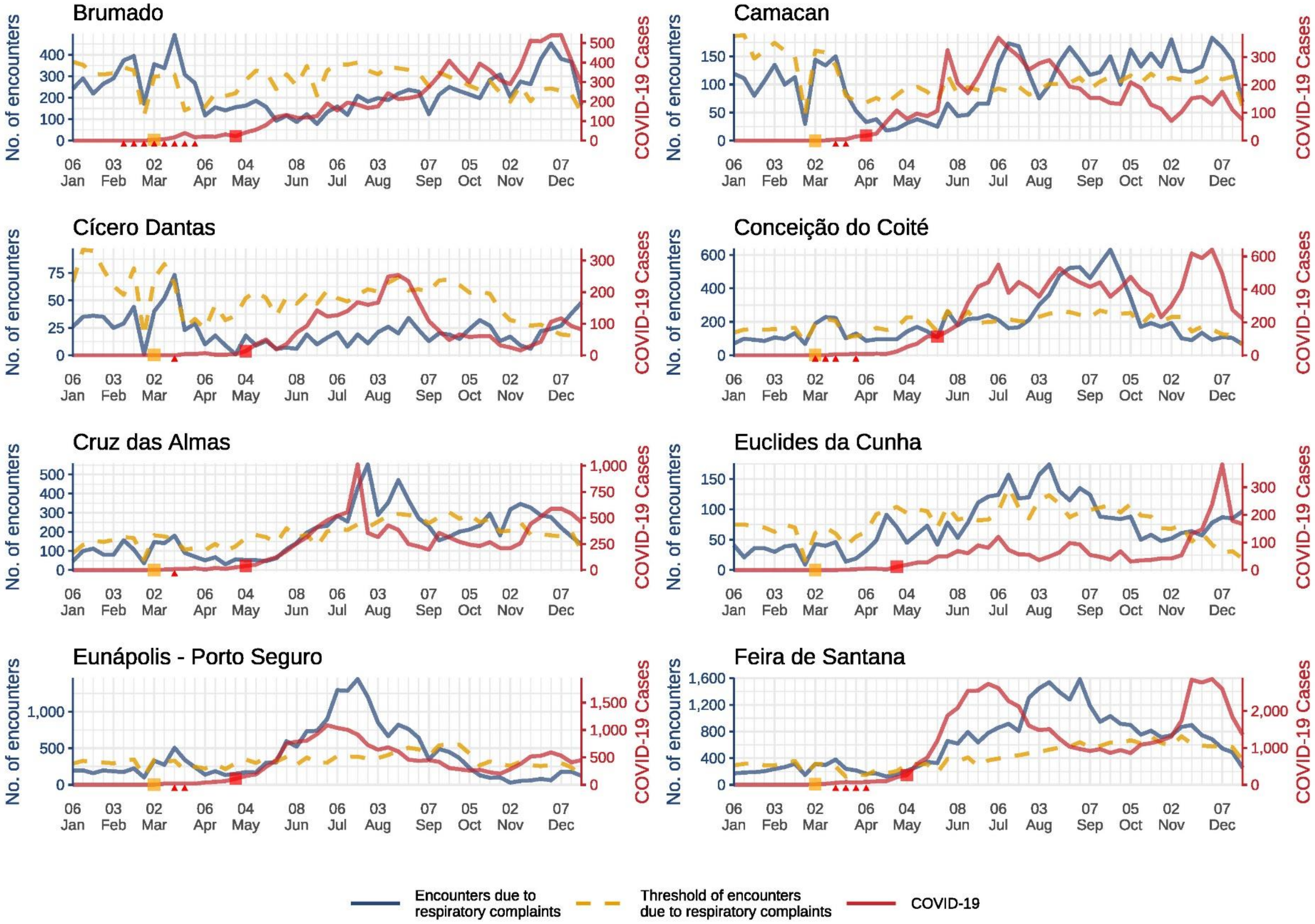

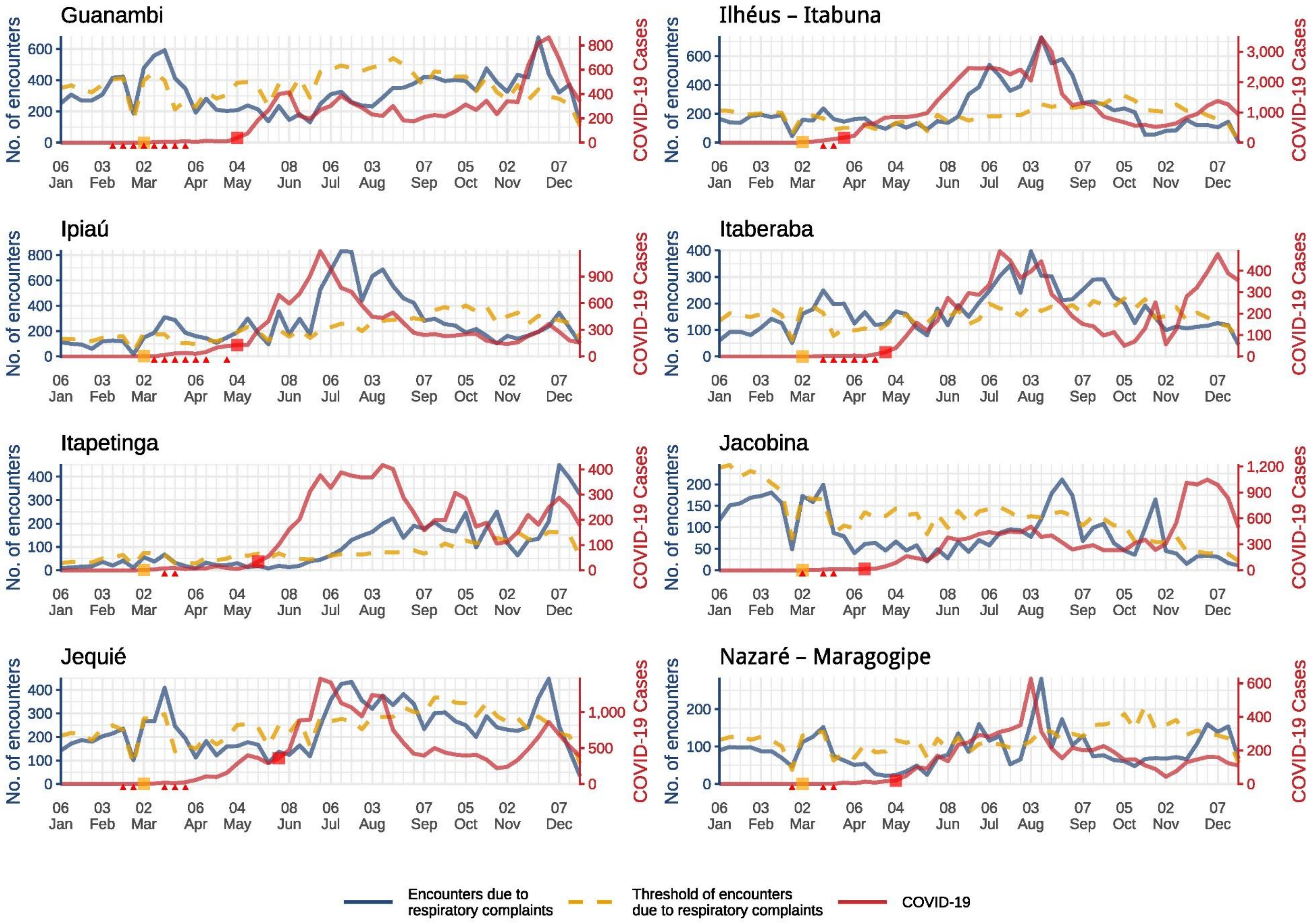
Guanambi
Ilhéus – Itabuna
Ipiaú
Itaberaba
Itapetinga
Jacobina
Jequié
Nazaré – Maragogipe
No. of encounters
COVID-19 Cases
06 Jan
03 Feb
02 Mar
06 Apr
04 May
08 Jun
06 Jul
03 Aug
07 Sep
05 Oct
02 Nov
07 Dec
Encounters due to respiratory complaints
Threshold of encounters due to respiratory complaints
COVID-19

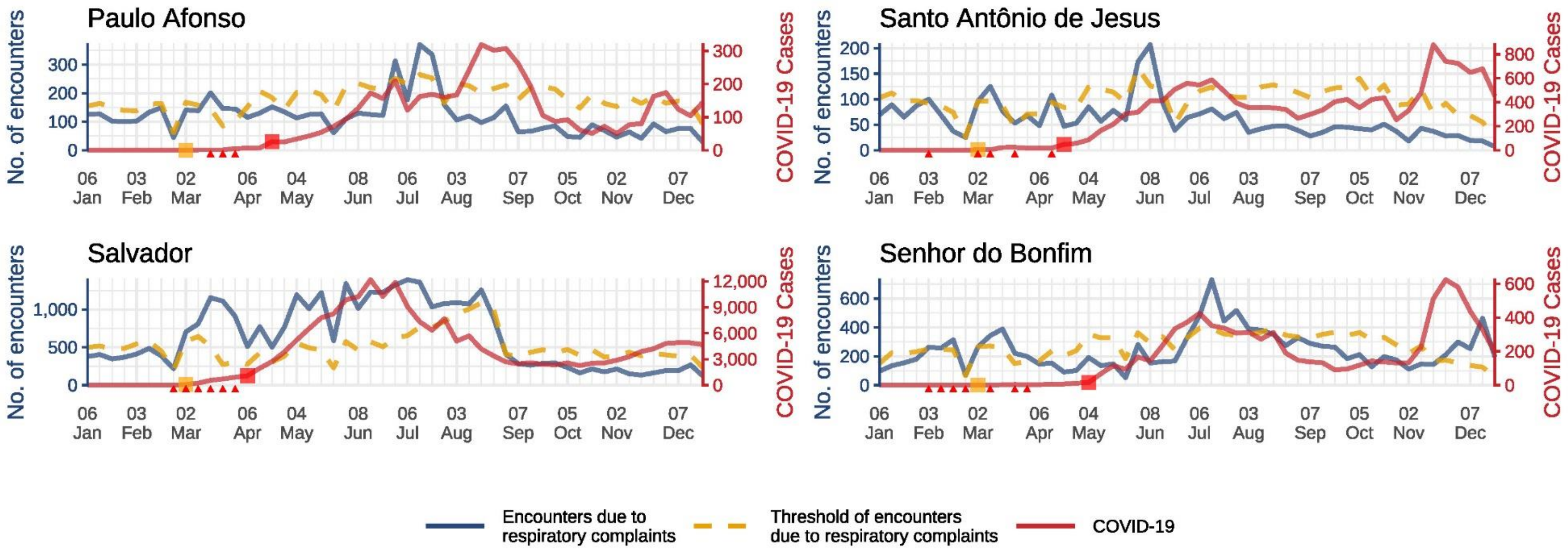
Paulo Afonso
Santo Antônio de Jesus
Salvador
Senhor do Bonfim
No. of encounters
COVID-19 Cases
06 Jan
03 Feb
02 Mar
06 Apr
04 May
08 Jun
06 Jul
03 Aug
07 Sep
05 Oct
02 Nov
07 Dec
Encounters due to respiratory complaints
Threshold of encounters due to respiratory complaints
COVID-19

Figure S3: Weekly syndromic surveillance signals from PHC and weekly COVID-19 cases from the three largest cities in Bahia 2020. The yellow square indicates the week of the first COVID-19 case confirmed in Bahia. The red square indicates the first week of the rapid growth of COVID-19 cases based on piecewise linear regression. The red triangles denote weeks with excess encounters due to respiratory complaints prior to the rapid growth of COVID-19 cases.

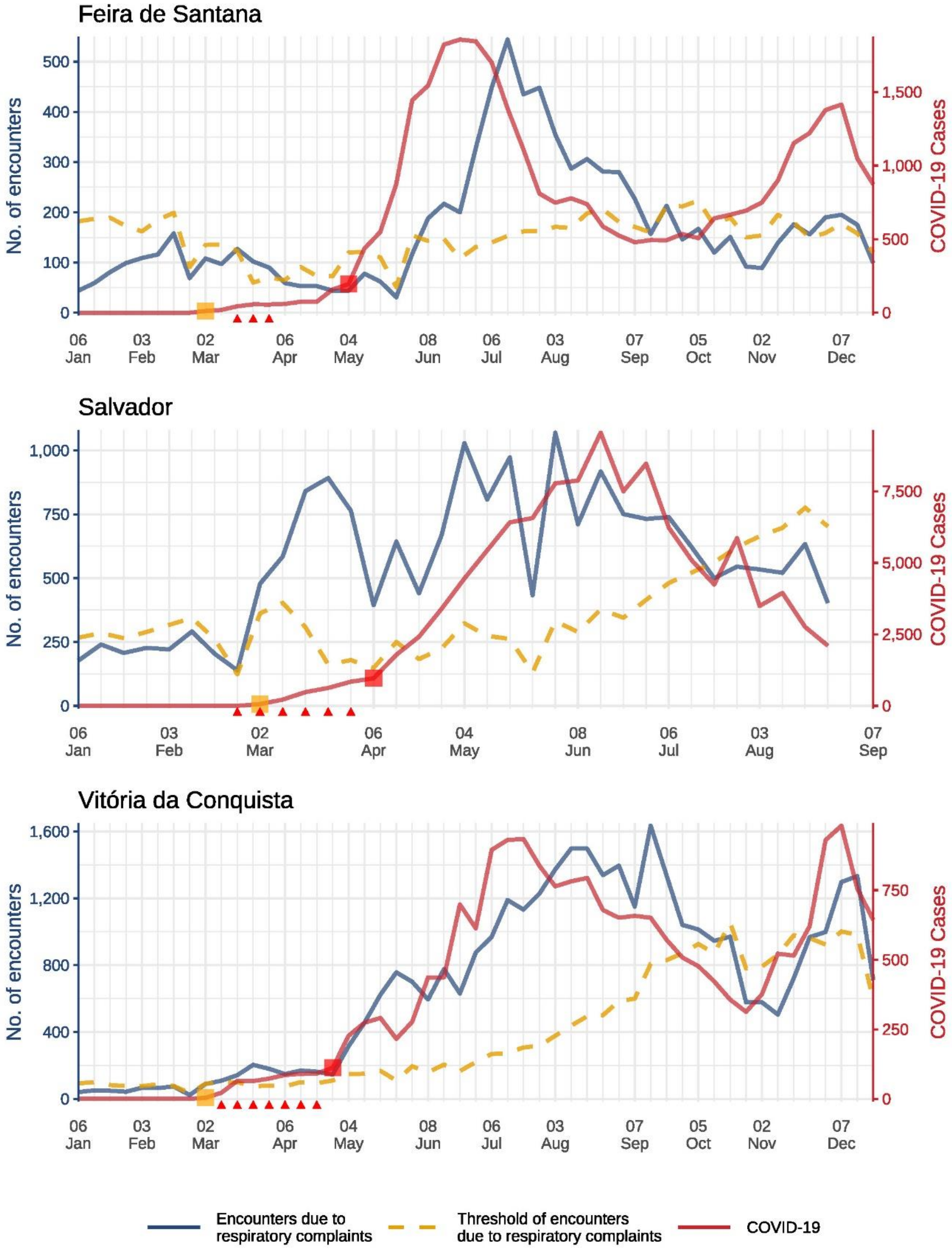

Figure S4: Fortnight number of hospitalisations from Severe Acute Respiratory Syndrome from the selected regions in 2020 stratified by diagnosis.

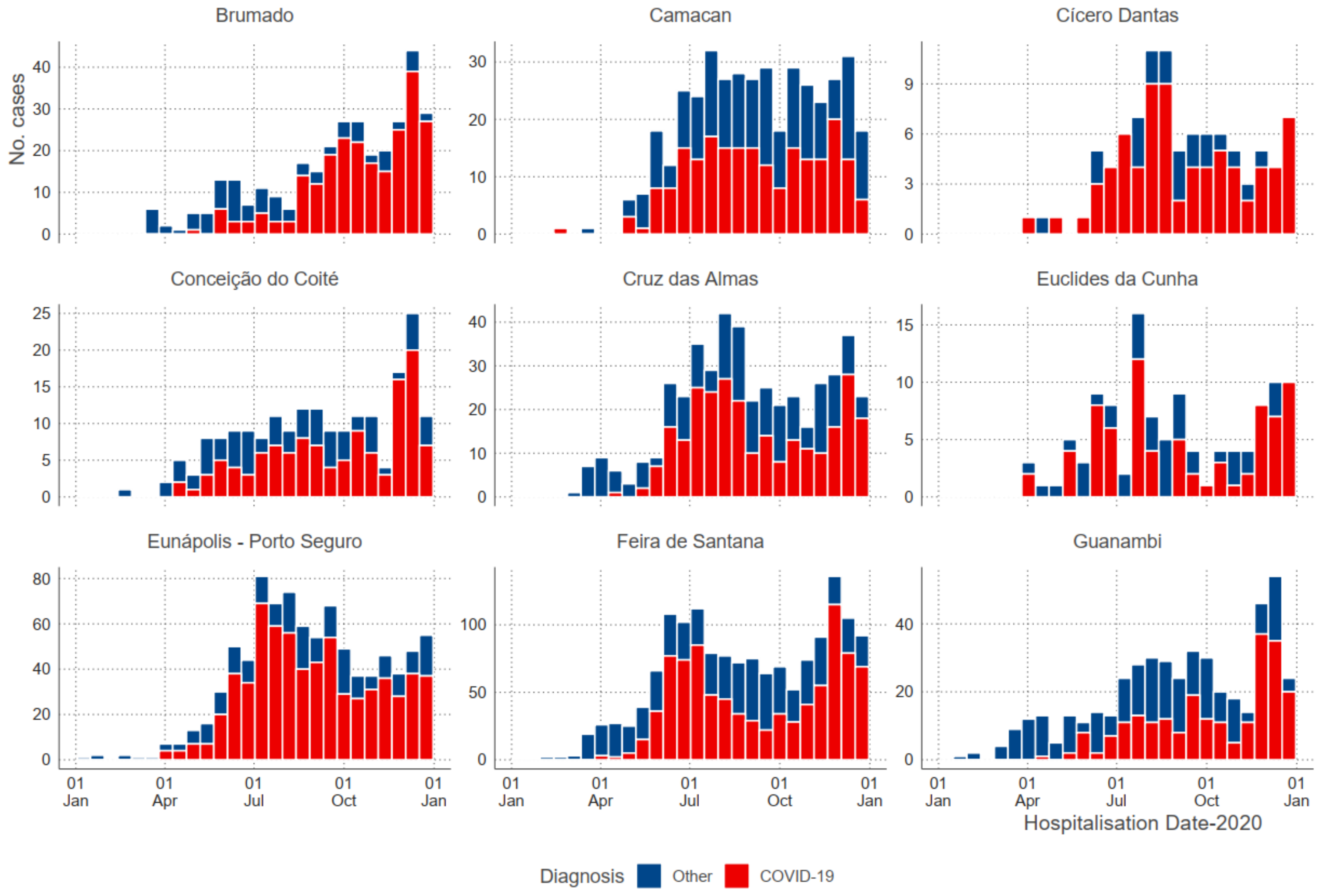

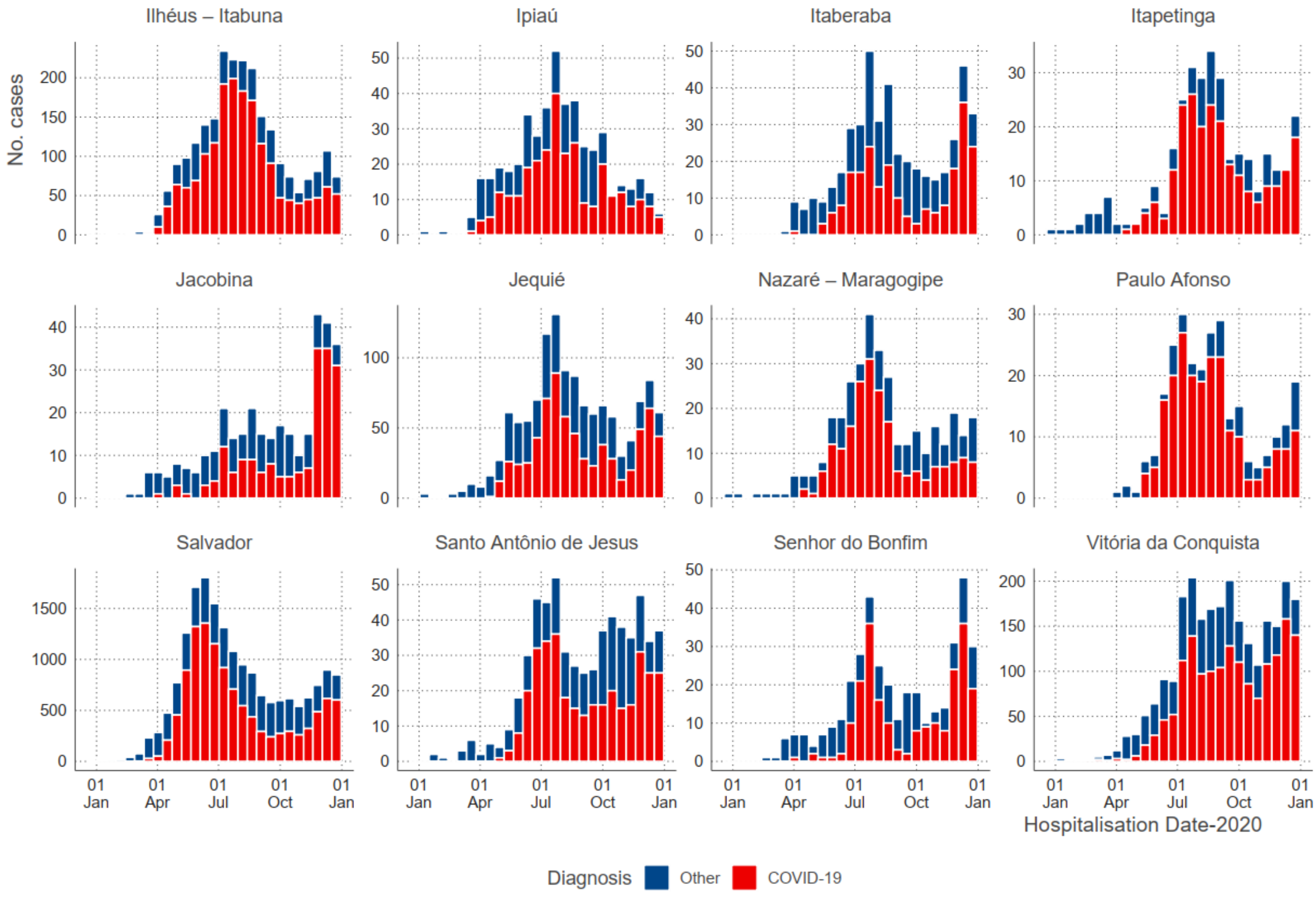
Ilhéus – Itabuna
Ipiaú
Itaberaba
Itapetinga
Jacobina
Jequié
Nazaré – Maragogipe
Paulo Afonso
Salvador
Santo Antônio de Jesus
Senhor do Bonfim
Vitória da Conquista
No. cases
01 Jan
01 Apr
01 Jul
01 Oct
01 Jan
Hospitalisation Date-2020
Diagnosis
Other
COVID-19

Figure S5: Example of piecewise regression and COVID-19 cases to define the breakpoints. Red line denotes COVID-19 cases, and the black line is the piecewise linear regression.

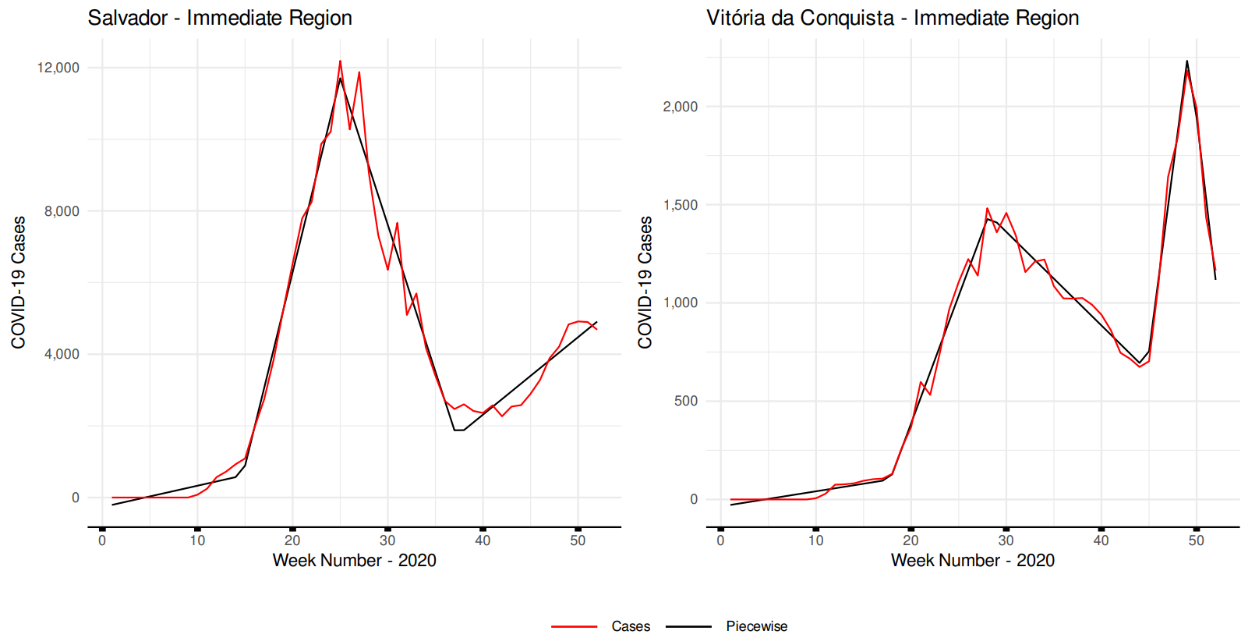

Supplement: Online Supplementary Document [file jogh-13-04124-s001.pdf]
